# Environmental risk of unintentional injuries at home for children aged 0–6 years in the urban area of Mianyang, China: A cross-sectional investigation

**DOI:** 10.1371/journal.pone.0336573

**Published:** 2025-12-09

**Authors:** Xiaoyu Li, Xiaoyue Tang, Fengqiong Jiang, Chunhua Zhang, Hongyan Chen, Rongxiang Zhao

**Affiliations:** 1 Mianyang Central Hospital, School of Medicine, University of Electronic Science and Technology of China, Mianyang, Sichuan, China; 2 Hi-Tech Zone Hospital for Women and Children, West China Second University Hospital, Sichuan University, Chengdu, China; Northern Border University, SAUDI ARABIA

## Abstract

To assess the environmental risk levels and factors associated with unintentional injuries in children’s (0–6 years) homes in Mianyang, China, this cross-sectional study used stratified random sampling and surveyed parents through an online questionnaire from April to June 2024. The survey comprised two parts: demographic information and an environmental scale that assessed unintentional injury risks at home. Family unintentional injury environmental scale scores are presented as median (interquartile range) and were analyzed using the Mann–Whitney rank-sum and Kruskal–Wallis H tests. Influencing factors were examined with a multiple linear regression model. Four hundred and five parents participated in the survey. The overall score for the environmental assessment was 92 (71–98.5) points, with 248 families (61.2%) classified as high risk. Significant differences were observed across demographics: families with children aged 1–3 years had the highest scores (93 [70.5, 99] points; H = 6.061, *P* < 0.05) compared with families with children <1 and 4–6 years. Families with grandparents as primary caregivers scored higher (94.5 [88.0, 100.0] points; H = 15.194, *P* < 0.001) than non-grandparent caregivers; caregivers with primary school education or less had the highest scores (95.0 [91.0, 99.0] points; H = 39.978, *P* < 0.001). Children with a history of unintentional injuries also had higher scores (94.0 [85.0, 99.0] points; Z = −3.219, *P* < 0.001) than those without. The χ^2^ values for risk level comparisons were 20.039, 24.206, 63.092, and 10.424, respectively (all *P* < 0.05). Multiple regression identified residence area, child age, caregiver education, training on injury prevention, and history of injuries as independent factors. Overall, home environments in the urban area of Mianyang predominantly pose a high risk of unintentional injury, with scores varying by demographic characteristics. Enhancing safety education for targeted groups and addressing potential hazards are crucial for reducing unintentional injuries among children.

## Introduction

Children represent a high-risk population for unintentional injuries. The World Health Organization has indicated that unintentional injuries are the leading cause of death among children aged 5–18 years [1]. Statistics further reveal that unintentional injuries account for one-third of annual fatalities in children and adolescents [2] and are the predominant cause of death among children aged 0–14 years in China [3]. Unintentional injuries not only significantly affect the daily lives of children and their families but may also lead to physical disabilities [4]. This profoundly impacts the physical and mental development of children and imposes a substantial burden on families and society. Studies have shown that the cost of unintentional injuries in children is estimated to range from USD 516,938 to USD 9,550,704 [5].

Children spend a significant portion of their formative years at home, making the home environment the most common location for unintentional injuries [6–8]. Studies conducted in China have indicated that unintentional home injuries account for 52.11%–58.9% of all unintentional injuries [4,9]. Furthermore, Pathak et al., reported that majority of the unintentional injuries in Nepal occurred at home (54.5%) [10]. An unsafe home environment is recognized as the primary risk factor for the occurrence of unintentional injuries in children [5], and the incidence of unintentional home injuries is directly correlated with home environment conditions [11,12].

Among children of different age groups, those aged 0–6 years exhibit a particularly high incidence of unintentional injuries [13,14]. In this age group, unintentional home injuries are the leading causes of both death and disability [15]. However, research focusing on this demographic is limited. Existing studies have primarily concentrated on the clinical and epidemiological characteristics of unintentional injuries, with insufficient attention given to interventions such as prevention and risk assessment. Research shows that the incidence of unintentional injuries among children in Mianyang City is 32.99% [16], with a mortality rate of 36.3% [17]. These rates are significantly higher than the incidence and mortality of accidental injuries among children in other areas of the province.

Therefore, this study aimed to assess the environmental risk levels associated with unintentional home injuries among children aged 0–6 years in the urban area of Mianyang, China. Specifically, it sought to answer the following questions: (1) What is the risk level of the home environment for unintentional injuries among children in urban Mianyang? (2) How do home environmental risk scores for unintentional injuries vary among families with different demographic characteristics? (3) What factors influence the risk of unintentional injuries in the home environment for children? Through this investigation, we aim to deepen the understanding of current environmental safety conditions and their influencing factors. Our findings will provide a reference for developing preventive strategies against unintentional injuries among children and lay a foundation for future intervention studies, ultimately contributing to a reduction in unintentional home injuries in this population.

## Materials and methods

### Study design

This was a cross-sectional study. The Strengthening the Reporting of Observational Studies in Epidemiology (STROBE) guidelines were followed.

### Participants

From April 26 to June 28, 2024, one preschool and one community hospital were selected from each of the three districts in the Mianyang urban area of China. Each district included both urban centers and township units. These sites were used to survey parents of children aged 0–6 years. The inclusion criteria were as follows: (1) having children aged 0–6 years; (2) being a long-term resident in the Mianyang urban area (≥1 year); and (3) voluntary participation. The exclusion criteria were as follows: (1) cognitive impairment or communication disorder and (2) inability to complete the questionnaire. The study was conducted in accordance with the Declaration of Helsinki and was approved by the Ethics Committee of Mianyang Central Hospital (approval no.: S20240325-01). All participants provided written informed consent prior to their participation in the study.

### Survey content and methods

#### Questionnaire survey.

The questionnaire included two components: (1) general data, consisting of demographic information on the child and parents, such as the child’s age, sex, residential address, history of unintentional injuries, the main caregiver’s educational level, and whether the main caregiver had received training on unintentional injuries; and (2) the unintentional home injury environmental risk score, derived from the China Urban Area 0–6-year-old Children’s Home Unintentional Injury Environment Scale developed by Wang et al. [18]. Permission for use of this scale was obtained from Dr. Wang Dong via email correspondence. The scale consists of 54 items across six dimensions: 15 items on falls, 12 on external force injuries, 12 on burns, 7 on poisonings, 6 on foreign body injuries, and 2 on animal-related injuries. Each item is rated on a 1–5 scale, corresponding to “Completely true,” “Mostly true,” “Somewhat true,” “Mostly not true,” and “Completely not true.” Items not applicable are scored 0. The maximum total score is 270, with higher scores indicating greater risk. Home environments are classified as low risk (<71 points), moderate risk (71–88 points), or high risk (≥89 points). Using this scale, the research team conducted a preliminary survey in the urban areas of Changsha to assess home environments for unintentional injuries among children aged 0–6 years. The results showed a split-half reliability coefficient of 0.82 and a Cronbach’s alpha of 0.87 for the total scale. Pearson correlation coefficients between the individual dimensions and the total scale were >0.5, while those among various dimensions were ≤0.6, indicating good reliability and validity.

#### Sample size and sampling method.

Based on the calculation formula of 5–20 times the number of items (54 in this study) [19] and accounting for a 15% disqualification rate, the minimum required sample size was 318 participants. For children aged <3 years, three community health centers were randomly selected from each of the three districts using stratified random sampling. Subsequently, children were selected from the age groups (<28 days, 28 days to <1 year, and 1 to <3 years) through stratified convenience sampling, and their parents were included as participants. For children aged 3–6 years, multi-stage stratified cluster random sampling was employed. In the first stage, three preschools were randomly selected from each district. In the second stage, one class was randomly selected from each grade level within each preschool, and their parents were included as participants.

#### Survey methods.

Participants were recruited via WeChat QR codes from April 26 to June 28, 2024. Data collection occurred concurrently with recruitment. No exposure or follow-up was involved. The survey questionnaire was distributed through Wenjuanxing, a Chinese online survey platform that generates a QR code for access. Trained researchers administered the survey and ensured proper distribution and collection. For children aged <3 years, approval was obtained from community health centers before survey administration. With assistance from healthcare personnel at these centers, the survey was administered to parents attending child healthcare or vaccination services. Research personnel provided on-site guidance for completing the questionnaire. For parents of the selected kindergarten students, the online survey allowed convenient access without requiring physical presence, thereby increasing participation. Approval was also obtained from kindergarten teachers before the survey. Teachers distributed a QR code linked to the questionnaire, enabling parents to complete it via the WeChat app (WeChat, Shenzhen, China). Research personnel provided assistance via phone calls or WeChat for any queries related to questionnaire completion. Before the survey, participants received detailed explanations of the study’s purpose, content, and instructions. Participants accessed the survey interface by scanning a QR code linked to Wenjuanxing. They completed the online questionnaire after reading the informed consent form and selecting the “I agree” checkbox to indicate consent. During questionnaire development on Wenjuanxing, a setting restricted submissions to one per IP address, preventing multiple submissions by the same respondent.

### Quality control

Measures to reduce potential biases included: (1) stratified random sampling with multistage cluster sampling to minimize selection bias; (2) inclusion of households with ≥1 year of permanent residency to reduce mobility-related bias; (3) use of rigorously validated instruments to ensure measurement reliability and validity; (4) standardized training of researchers to provide neutral guidance during questionnaire completion, preventing response distortion; and (5) an explicit anonymity declaration in the preamble to mitigate underreporting due to concerns about negative evaluation.

To ensure the credibility of survey results, additional measures were implemented: (1) key points for questionnaire completion were outlined in the introduction section of the questionnaire; (2) research personnel provided on-site or online guidance for parents during completion; and (3) questionnaires with a response time of <3 min were excluded from analysis. Mandatory questions and logic-jump functions were embedded to ensure completeness, requiring participants to answer all items before submission.

### Statistical analysis

To control for potential confounding factors, we prespecified covariates including child age, caregiver characteristics, and the geographic region. All prespecified variables were retained in the multiple linear regression model using the forced-entry method, regardless of statistical significance in univariate analyses. Collinearity diagnostics confirmed acceptable tolerance (>0.4) and variance inflation factors (VIF < 2.5), indicating no substantial multicollinearity (VIF < 5 considered acceptable).

Children were categorized into three age groups based on their developmental stages: < 1 year, 1–3 years, and 4–6 years. Primary caregivers were classified as parents, paternal/maternal grandparents, and nannies/others. Educational attainment of caregivers was grouped as elementary school or below, junior high school/high school, and university or above. Residence was classified by administrative division into District A, District B, and District C. The specific district names have been anonymized to protect participant privacy.

Data were exported from Wenjuanxing and imported into SPSS 26.0 (IBM Corp., Armonk, NY, USA) for analysis. Normality testing yielded *P* < 0.001, indicating a skewed distribution. Unintentional home injury environmental risk scores were expressed as median [interquartile range] (M [Q1, Q3]), intergroup comparisons were performed using the Mann–Whitney U test or Kruskal–Wallis H test. Categorical variables were presented as frequencies and percentages, with comparisons using the Chi-square test. Factors influencing the unintentional home injury environmental risk score were analyzed using multiple linear regression, with the total unintentional home injury environmental risk score as the dependent variable. Independent variables included child age, caregiver type, educational level of the primary caregiver, area of residence, caregiver training in unintentional injury prevention, and child’s history of unintentional injuries. Two-sided tests were applied with a significance level of α = 0.05.

## Results

### Basic sociodemographic characteristics of the participants

A total of 418 participants completed the survey, yielding a response rate of 100%. After excluding 13 questionnaires due to response times <3 min or missing values, 405 participants were included in the final analysis, resulting in an effective response rate of 96.99%. The proportions of participants residing in District A, District B, and District C, were 49.9%, 26.7%, and 23.5%, respectively. Children aged <1 year accounted for 6.91% of the participants, whereas those aged 1–3 and 4–6 years comprised 40.74% and 52.35%, respectively. Overall, 48.1% of children had a history of unintentional injuries, and 51.9% had not. The primary caregivers were parents (53.6%), paternal/maternal grandparents (41.0%), and nannies/others (5.4%). Caregiver educational levels were as follows: elementary school or below, 36.8%; junior high school or high school, 36.0%; and university education or higher, 27.2%. In addition, 65.9% of caregivers had received training on unintentional injury prevention, while 34.1% had not (S1 Table).

### Reliability and validity testing of the unintentional home injury environmental risk scale

Cronbach’s alpha for the total scale was 0.956, with dimension values ranging from 0.453 to 0.899 (S2 Table). Exploratory factor analysis showed Kaiser–Meyer–Olkin values of 0.943 for the total scale and 0.880, 0.935, 0.874, 0.858, 0.795, and 0.500 for the six dimensions (falls, external force injuries, burns, poisonings, foreign body injuries, and animal-related injuries, respectively). Bartlett’s test of sphericity was significant for all dimensions (*P* < 0.001). The cumulative variance explained by the total scale common factors was 64.709%, with all item factor loadings >0.30. The cumulative variance explained by the common factors of the six dimensions were 53.888%, 48.178%, 58.378%, 45.427%, 50.041%, and 64.738%, respectively, with all item factor loadings also >0.30.

### Unintentional home injury environmental risks for children aged 0–6 years

The median total score for unintentional home injury environmental risk was 92 (71, 98.5), indicating a high-risk level. The minimum and maximum scores were 53 and 211, respectively. Median (interquartile range) for each dimension were: falls, 26 (19, 30); external force injuries, 20 (15, 23); burns, 19 (15, 22); poisonings, 12 (9, 14); foreign body injuries, 10 (8, 12); and animal-related injuries, 3 (2, 4). The percentage scores for these dimensions ranged from 30.0% to 34.7%, with falls exhibiting the highest percentage and animal-related injuries the lowest (Table 1). Based on total scores, 100 families (24.7%) were classified as low risk, 57 (14.1%) as moderate risk, and 248 (61.2%) as high risk (S1 Fig).

**Table 1:** Assessment results of home unintentional injuries environment among children aged 0–6 years in the urban area.

Item	Number of Items	Score *M* (*Q1*, *Q3*)	Maximum Possible Score	Score Percentage (%)
Total scale score	54	92 (71, 98.5)	270	34
Falls	15	26 (19, 30)	75	34.7
External force injuries	12	20 (15, 23)	60	33.3
Burns	12	19 (15, 22)	60	31.7
Poisonings	7	12 (9, 14)	35	34.3
Foreign body injuries	6	10 (8, 12)	30	33.3
Animal-related injuries	2	3 (2, 4)	10	30

### Comparison of unintentional home injury environmental assessment scores among populations with different characteristics

Scores and risk-level distributions differed significantly by age group, type of main caregiver, educational level of main caregiver, and history of unintentional injuries (all *P* < 0.05). The highest score, 93 (70.50, 99) points (H = 6.061, *P* = 0.048), was observed among children aged 1–3 years. Children cared for primarily by paternal or maternal grandparents had higher scores than those cared for by parents or nannies/others, with a median score of 94.50 (88.00, 100.00) points (H = 15.194, *P* < 0.001). Main caregivers with an educational level of elementary school or below had the highest scores, 95.00 (91.00, 99.00) points (H = 39.978, *P* < 0.001), compared with caregivers with higher educational levels. Children with a history of unintentional injuries scored higher (94.00 [85.00, 99.00] points [Z = −3.219, *P* < 0.001]), than those without such a history (Table 2). Comparison of risk levels showed χ^2^ values of 20.039, 24.206, 63.092, and 10.424, respectively, with all *P*-values <0.05 (Table 3).

**Table 2:** Comparison of home unintentional injury environmental risk scores by population characteristics (n = 405).

Variable	Number of Participants (Percentage [%])		Score *M* (*Q1*, *Q3*)	*Z/H* Value	*P*-value
**Area of residence**				1.736	0.420
District A	202	49.9	90.50 (67.00, 99.00)		
District B	108	26.7	93.00 (79.75, 98.00)		
District C	95	23.4	93.00 (76.00, 98.00)		
**Age of child**				6.061	0.048
< 1 year	28	6.9	77.00 (55.50, 94.75)		
1–3 years	165	40.7	93 (70.50, 99)		
4–6 years	212	52.4	92.50 (75.00, 98.70)		
**Main caregiver**				15.194	0.001
Parent	217	53.6	90.00 (67.00, 97.00)		
Paternal/maternal grandparent	166	41.0	94.50 (88.00, 100.00)		
Other	22	5.4	70.00 (88.00, 100.00)		
**Educational level of the main caregiver**				39.978	<0.001
Elementary school	149	36.8	95.00 (91.00, 99.00)		
Junior high school/high school	146	36.0	91.00 (68.00, 99.00)		
University or above	110	27.2	74.00 (58.00, 95.00)		
**Received training on unintentional injury prevention**				1.606^a^	0.108
Yes	267	65.9	92.00 (68.00, 98.00)		
No	138	34.1	93.00 (74.00, 100.25)		
**History of unintentional injuries in the child**				−3.219^a^	0.001
Yes	195	48.1	94.00 (85.00, 99.00)		
No	210	51.9	90.00 (65.00, 97.00)		

^a^denotes values obtained by the Mann–Whitney U test. All other values were obtained by the Kruskal–Wallis H test.

**Table 3:** Comparison of home unintentional injury environmental risk levels by population characteristics.

Variable	Risk Level			*χ*^*2*^ Value	*P*-value
	Low risk (n = 100)	Moderate risk (n = 56)	High risk (n = 249)		
**Area of residence**				3.139	0.535
District A	57 (28.2%)	28 (13.9%)	117 (57.9%)		
District B	22 (20.4%)	14 (13.0%)	72 (66.7%)		
District C	21 (22.1%)	14 (14.7%)	60 (63.2%)		
**Age of child**				20.039	0.001
< 1 year	15 (53.6%)	5 (17.9%)	8 (28.5%)		
1–3 years	32 (19.4%)	18 (10.9%)	115 (69.7%)		
4–6 years	53 (25.0%)	33 (15.6%)	126 (59.4%)		
**Main caregiver**				24.206	<0.001
Parent	63 (29.0%)	37 (17.1%)	117 (53.9%)		
Paternal/maternal grandparent	26 (15.7%)	17 (10.2%)	123 (74.1%)		
Other	11 (50.0%)	2 (9.1%)	9 (40.9%)		
**Educational level of the main caregiver**				63.092	<0.001
Elementary school	14 (9.4%)	12 (8.0%)	123 (82.6%)		
Junior high school/high school	37 (25.3%)	21 (14.4%)	88 (60.3%)		
University or above	49 (44.5%)	23 (20.9%)	38 (34.6%)		
**Received training on unintentional injury prevention**				1.622	0.444
Yes	71 (26.6%)	37 (13.9%)	159 (59.6%)		
No	29 (21.0%)	19 (13.8%)	90 (65.2%)		
**History of unintentional injuries in child**				10.424	0.005
No	62 (62.0%)	26 (46.4%)	107 (43.0%)		
Yes	38 (38.0%)	30 (53.6%)	142 (57.0%)		

### Influencing factors of home unintentional injury environmental risk scores for children aged 0–6 years in the urban area

Multiple linear regression analysis was performed with the total home unintentional injury environmental risk score as the dependent variable. Variables for the multiple regression model were selected based on their significance in the univariate analysis (Mann–Whitney and Kruskal–Wallis tests, *P* < 0.05). In addition, previous studies have reported that safety education influences the occurrence of accidental childhood injuries [20] and that regional differences exist in injury occurrence [21]; therefore, we included the variables “area of residence” and “receipt of training on unintentional injury prevention by the main caregiver” in the multiple analysis based on clinical relevance, even though they were not significant in the univariate analyses. Independent variables included child age, type of main caregiver, educational level of the main caregiver, area of residence, receipt of training on unintentional injury prevention by the main caregiver, and the presence or absence of a history of unintentional injuries in the child. The results indicated that area of residence, child age, educational level of the main caregiver, receipt of unintentional injury-prevention training, and a history of unintentional injuries in the child were independent influencing factors of the environmental risk score. Specifically, residing in the District B, being aged 1–3 years, having a main caregiver with low educational level, not receiving training on unintentional injury prevention, and having a history of unintentional injuries were associated with higher risk (Table 4).

**Table 4:** Multiple linear regression analysis of factors associated with home unintentional injury environmental risk scores among children aged 0–6 years in the urban area.

Variable	Unstandardized Coefficient		Standardized Coefficient	*t-*value	*P*-value
	B (95%CI) value	SE value	*β*		
Constant	105.414 (102.751–108.078)	1.359		77.578	<0.001
Area of residence	2.369 (1.934–2.804)	0.222	0.055	10.676	<0.001
Age	−1.438 (−2.011 to −0.865)	0.292	−0.025	−4.920	<0.001
Main caregiver	0.113 (−0.491 to 0.717)	0.308	0.002	0.367	0.714
Educational level of main caregiver	−7.928 (−8.389 to −7.468)	0.235	−0.177	−33.762	<0.001
Receipt or non-receipt of training on unintentional injury prevention	4.040 (3.313–4.767)	0.371	0.055	10.892	<0.001
History of unintentional injuries in the child	9.557 (8.860–10.254)	0.355	0.136	26.886	<0.001
R^2^			0.063		
Adjusted R^2^			0.063		
F			422.710		0.001

## Discussion

This cross-sectional study included 405 parents from urban Mianyang to evaluate home environments for unintentional injuries. The median total score for the home environment risk assessment was 92 (71.0–98.5), indicating an overall high-risk level. Significant differences in scores and risk levels were observed across families with different demographic characteristics. Multiple analysis identified the residential area, child’s age, caregiver’s educational level, caregiver training on unintentional injury prevention, and the child’s history of unintentional injuries as independent influencing factors of risk scores. By focusing on high-incidence age groups and locations, we conducted a pre-intervention assessment of the home environment risk levels. These findings expand domestic research on child unintentional injury prevention and provide a valuable reference for policymakers to develop targeted prevention strategies.

### Types of main caregivers and incidence rates of unintentional injuries among children aged 0–6 years in urban areas

In this study, the incidence rate of unintentional injuries among children was 48.1%. This rate was lower than those reported by Chen et al. [12] and Dong et al. [22], but higher than those reported by Zhang et al. [19], Yu et al. [23], Sun et al. [24], Luo et al. [25], and Shen et al. [14]. Specifically, Chen et al. [12] reported an incidence of 78.7% among children aged 0–7 years in Wuhan, and Dong et al. [22] reported 48.90% among left-behind children (by migrant parents) in four ethnic minority autonomous counties across the Western Chinese provinces of Guangxi, Yunnan, Sichuan, and Guizhou. Both studies involved rural populations, where greater environmental risk exposure may have contributed to the higher rates. In contrast, Zhang et al. [19] reported incidence rates of 35.2%–41.1% among elementary and junior high school students in Yunnan, and Yu et al. [23] found an incidence of 15.53% among adolescents in Beijing and Guangdong. In provincial-level urban areas, the incidence among children aged 0–6 years was reported as 9.0% in Qingdao City [24], 16.9% in Urumqi City [25], and 10.8% in Shanghai City [14], all lower than the rate found in the present study. These discrepancies may be explained by (1) age-related differences: compared with children aged 0–6 years, older students have stronger self-protection awareness and skills; (2) regional factors: area-specific differences, including economic development, strongly affect injury prevention.

In this study, parents accounted for 53.6% of primary caregivers, while the remaining 46.4% were grandparents or other relatives. Among caregivers, 72.8% had a high school education or lower, and 34.1% had not received training related to unintentional injuries. In contrast, Dong et al. [22] and Zhang et al. [19] reported that non-parent caregivers constituted 76.40% to 82.78% of primary caregivers. The high proportion of non-parent primary caregivers, coupled with their generally low educational levels and limited opportunities for training, underscores the need for continuous, effective interventions to improve child safety. Training in unintentional injury prevention should extend beyond parents to include grandparents and other relatives.

### Home environments of children aged 0–6 years in urban areas require improvement

The total unintentional home injury environmental assessment score for children aged 0–6 years in urban areas was 92 (71, 98.5), higher than that reported by Wang et al. in Changsha City, China (81.60–86.45) [18], indicating a high-risk level. Few studies have assessed the home unintentional injury environments in China. In 2011, the Alliance for Safe Children, in collaboration with partner organizations, released the Child Safe Home Checklist [26]; however, its application has not yet been reported. Chen et al. investigated home and playground factors associated with unintentional injuries in children in Wuhan City, China, but did not provide quantitative or qualitative data on environmental risks [12]. Shields et al. [27] developed the Child Housing Assessment for a Safe Environment (CHASE) tool, comprising 25 items across 12 dimensions, and applied it to assess 142 families in Baltimore, Maryland, USA. Findings showed that only 54% to 59% of families passed the safety assessment. In the present study, high-risk families comprised 61.2%, while moderate- and low-risk families together accounted for 38.8%.

Home environmental risk was highest for falls and lowest for animal-related injuries, consistent with reported incidence patterns of unintentional injuries in children [24,28,29]. However, another study found that animal-related injuries represented the largest proportion of unintentional injuries among children aged 0–5 years [30]. This discrepancy may be attributed to the fact that the latter study was based on populations seeking care at sentinel hospitals. Such populations often have heightened awareness of the need for rabies treatment and vaccination, which may increase medical consultations for animal-related injuries compared with other injury types.

In summary, both the total home environmental risk score and the proportion of high-risk families were relatively high among children in the Mianyang urban area. Thus, efforts to reduce home environmental risks, particularly falls, remain urgently needed.

### Influencing factors of the home unintentional injury environmental risks for children aged 0–6 years in the urban area

Scores and risk levels varied significantly by child age, caregiver type, educational level of main caregivers, and history of unintentional injuries. Multiple linear regression analysis identified the area of residence, child age, main caregiver educational level, training on unintentional injury prevention, and history of unintentional injuries in the child as independent factors influencing the risk score. Families in B and C Districts had higher proportions of high-risk scores than those in the District A, possibly because the former districts include more township administrative units. Similarly, previous studies have shown that rural areas and rural-urban fringes have higher incidences of unintentional injuries than urban centers [10,18,21,22]. Scores were also higher among families in which the main caregiver had a lower educational level, possibly reflecting limited internet literacy and fewer opportunities to receive safety training. Consequently, these factors are likely to contribute to increased household environmental risk.

Additionally, a history of unintentional injuries in the child was associated with higher risk scores, suggesting that families with prior incidents did not effectively learn from past experiences or implement preventive measures. This highlights the importance of strengthening health education.

Although the regression model yielded a relatively low R^2^ value, this may reflect the complexity of factors influencing household injury risks. Unmeasured variables, such as family economic status or the number of children who were minors, may have contributed, and future studies should address these to improve predictive accuracy. Notably, several influencing factors identified in this study—child age, caregiver educational, safety training, area of residence, and caregiver type—align with previously reported determinants of unintentional childhood injuries [19–21,31].

### Recommendations for enhancing home environment safety

Children’s bodies and minds are not yet fully developed, resulting in a limited capacity to recognize risks and hazards [12]. Consequently, ensuring a safe home environment is of paramount importance. Based on the findings of this study, we propose several recommendations to enhance home environmental safety for children and prevent unintentional home injuries. First, management strategies should target key families by addressing the factors associated with unintentional home injuries in children aged 0–6 years. Key families are defined as those with the following characteristics: residing in townships, having a child aged 1–3 years, having a main caregiver with an educational level of elementary school or below, not having received training on unintentional injury prevention, and having a child with a history of such injuries. Second, dissemination of knowledge on unintentional injuries should be intensified among primary caregivers. Bhatta et al. and Stager et al. suggested that parents can enhance child safety by eliminating environmental risks or increasing access to safety equipment. However, caregivers often have limited knowledge of hazards and risk management and lack effective intervention tools [15,32]. Therefore, caregivers should be equipped with knowledge to recognize and address home environmental risks through the dissemination of scientific information. Lastly, promoting environmental management behaviors aimed at preventing unintentional home injuries is essential. Ma et al. emphasized that effective prevention should prioritize actionable measures and interventions within the home [33]. In addition, Bhatta et al. reported that home modifications reduced the incidence of such injuries [34]. Stewart et al. recommended the “Stay One Step Ahead” home safety intervention to strengthen environmental management. A key component of this program is the use of a home safety checklist by health personnel to systematically identify and eliminate risks [35]. Therefore, we recommend that caregivers use the China Urban Area 0–6-year-old Children’s Home Unintentional Injury Environment Scale as a checklist to regularly assess and address home safety risks, with support from community general practitioners or nurses.

### Limitations

This study has some limitations. First, its cross-sectional design precludes causal inference. Second, the online survey method prevented face-to-face guidance during questionnaire completion, potentially introducing a self-reporting bias from caregivers. To mitigate this, the research team provided detailed instructions in the questionnaire preface using simple, clear language. Third, because the data were collected exclusively from urban areas of Mianyang, the results may not be generalizable to other urban settings with different sociodemographic characteristics or to rural populations. Finally, the relatively small sample size may further limit generalizability. To address this, stratified sampling and multistage stratified cluster random sampling methods were applied. Future studies should incorporate cluster sampling and regional census approaches to improve representativeness and provide a more accurate assessment of unintentional injury risks in different household environments.

### Significance

This study assessed the risk levels of home environments for unintentional injuries among children living in urban areas of Mianyang, China, and analyzed the distribution of at-risk families and their influencing factors. The findings provide scientific evidence for policymakers to develop child safety measures, theoretical support for community nurses in designing and implementing safety education and training programs, and a basis for future intervention research on unintentional household injuries among children.

## Conclusions

This study revealed a high risk of unintentional home injuries to children in urban Mianyang. Assessment scores varied significantly across families with different characteristics, with independent influencing factors including sociodemographic and caregiver-related variables. Strengthening home safety education is particularly important for families living in townships, those with children aged 1–3 years, families where grandparents are the primary caregivers, caregivers with a high school education or below, children with a history of unintentional injuries, and caregivers lacking related training. Promoting the use of the environmental risk assessment scale for children aged 0–6 years may help identify and address safety risks, thereby reducing the incidence of unintentional home injuries.

## Supporting information

S1 Table Basic sociodemographic characteristics of the participants (n = 405). (DOCX)

S2 Table Reliability analysis of the home unintentional injury environmental risk scale for children aged 0–6 years in this study. (DOCX)

S1 Fig Distribution of the home unintentional injury risk levels in children (0–6 years). (TIF)

S1 File STROBE checklist. (DOCX)
