# Supplementary material for: Environmental risk of unintentional injuries at home for children aged 0–6 years in the urban area of Mianyang, China: A cross-sectional investigation
Source: PLoS One. 2025 Dec 9;20(12):e0336573. doi: 10.1371/journal.pone.0336573 (PMC12688114; doi:10.1371/journal.pone.0336573)
Supplement: S1 Table — (DOCX) [file pone.0336573.s001.docx]

**S1 Table**

| Variable | Number of participants | Percentage (%) |
| --- | --- | --- |
| **Area of residence** | | |
| District A | 202 | 49.9 |
| District B | 108 | 26.7 |
| District C | 95 | 23.4 |
| **Age of child** | | |
| <1 year | 28 | 6.9 |
| 1–3 years | 165 | 40.7 |
| 4–6 years | 212 | 52.4 |
| **Main caregiver** | | |
| Parent | 217 | 53.6 |
| Paternal/maternal grandparent | 166 | 41.0 |
| Other | 22 | 5.4 |
| **Educational level of main caregiver** | | |
| Elementary school | 149 | 36.8 |
| Junior high school/high school | 146 | 36.0 |
| University or above | 110 | 27.2 |
| **Receipt or non-receipt of training on knowledge related to unintentional injuries** | | |
| Yes | 267 | 65.9 |
| No | 138 | 34.1 |
| **History of unintentional injuries in child** | | |
| Yes | 195 | 48.1 |
| No | 210 | 51.9 |
