# Supplementary material for: Environmental risk of unintentional injuries at home for children aged 0–6 years in the urban area of Mianyang, China: A cross-sectional investigation
Source: PLoS One. 2025 Dec 9;20(12):e0336573. doi: 10.1371/journal.pone.0336573 (PMC12688114; doi:10.1371/journal.pone.0336573)
Supplement: S2 Table — (DOCX) [file pone.0336573.s002.docx]

**S2 Table**

| **Dimension** | **Number of Items** | **Cronbach’s Alpha** |
| --- | --- | --- |
| Falls | 15 | 0.806 |
| External force injuries | 12 | 0.899 |
| Burns | 12 | 0.848 |
| Poisonings | 7 | 0.780 |
| Foreign body injuries | 6 | 0.800 |
| Animal-related injuries | 2 | 0.453 |
| Total | 54 | 0.956 |
