# Supplementary figures and images for: Environmental risk of unintentional injuries at home for children aged 0–6 years in the urban area of Mianyang, China: A cross-sectional investigation
Source: PLoS One. 2025 Dec 9;20(12):e0336573. doi: 10.1371/journal.pone.0336573 (PMC12688114; doi:10.1371/journal.pone.0336573)

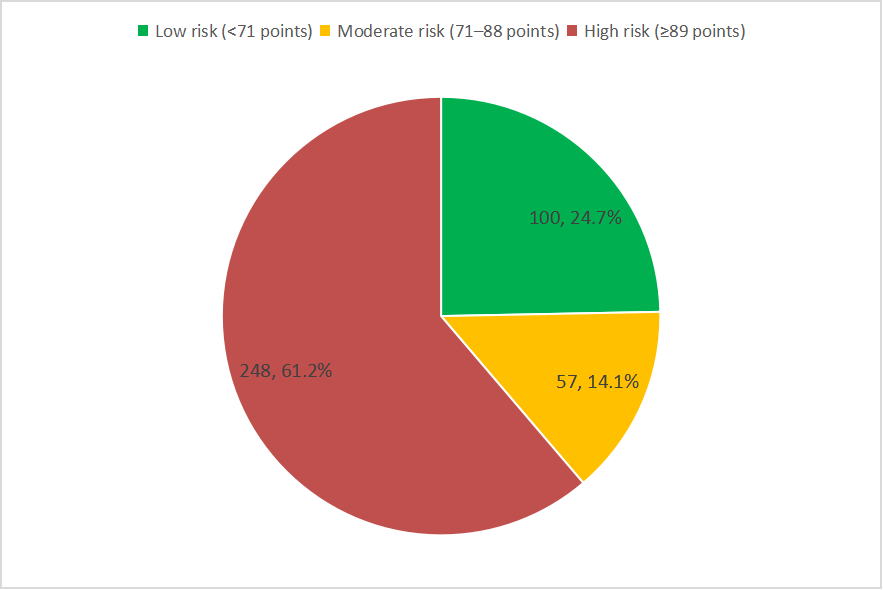

Supplement: S1 Fig — (TIF) [file pone.0336573.s003.tif]
