# Supplementary material for: Environmental risk of unintentional injuries at home for children aged 0–6 years in the urban area of Mianyang, China: A cross-sectional investigation
Source: PLoS One. 2025 Dec 9;20(12):e0336573. doi: 10.1371/journal.pone.0336573 (PMC12688114; doi:10.1371/journal.pone.0336573)
Supplement: S1 File — (DOCX) [file pone.0336573.s004.docx]

**S1 File**

|  | Item No | Recommendation | Reported on Page No |
| --- | --- | --- | --- |
| **Title and abstract** | 1 | (*a*) Indicate the study’s design with a commonly used term in the title or the abstract | Page 1: Title: “cross-sectional investigation”  Page 2, Line 23, Section: Abstract |
|  |  | (*b*) Provide in the abstract an informative and balanced summary of what was done and what was found | Page 2, Section: Abstract: The aim, methods, findings, and conclusions are reported |
| Introduction | | |  |
| Background/rationale | 2 | Explain the scientific background and rationale for the investigation being reported | Pages 3-4, Lines 47-65, Section: Introduction |
| Objectives | 3 | State specific objectives, including any prespecified hypotheses | Page 4, 75-85, Section: Introduction |
| Methods | | |  |
| Study design | 4 | Present key elements of study design early in the paper | Page 4, Lines 91-92, Section: Study design |
| Setting | 5 | Describe the setting, locations, and relevant dates, including periods of recruitment, exposure, follow-up, and data collection | Page 7-8, Lines 143-162, Section: Survey methods |
| Participants | 6 | (*a*) *Cohort study*—Give the eligibility criteria, and the sources and methods of selection of participants. Describe methods of follow-up  *Case-control study*—Give the eligibility criteria, and the sources and methods of case ascertainment and control selection. Give the rationale for the choice of cases and controls  *Cross-sectional study*—Give the eligibility criteria, and the sources and methods of selection of participants | Page 5, Lines 95-105, Section: Participants  Page 7, Lines 130-140, Section: Sample size and sampling method |
|  |  | (*b*) *Cohort study*—For matched studies, give matching criteria and number of exposed and unexposed  *Case-control study*—For matched studies, give matching criteria and the number of controls per case | *Not applicable - Cross-sectional study* |
| Variables | 7 | Clearly define all outcomes, exposures, predictors, potential confounders, and effect modifiers. Give diagnostic criteria, if applicable | Page 5, Lines 109-127, Section: Questionnaire survey |
| Data sources/ measurement | 8* | For each variable of interest, give sources of data and details of methods of assessment (measurement). Describe comparability of assessment methods if there is more than one group | Page 7, Lines 143-162, Section: Survey methods |
| Bias | 9 | Describe any efforts to address potential sources of bias | Pages 8-9, Lines 165-177, Section: Quality control |
| Study size | 10 | Explain how the study size was arrived at | Page 7, Lines 130-140, Section: Sample size and sampling method |
| Quantitative variables | 11 | Explain how quantitative variables were handled in the analyses. If applicable, describe which groupings were chosen and why | Page 9, Lines 186-191, Section: Statistical analysis |
| Statistical methods | 12 | (*a*) Describe all statistical methods, including those used to control for confounding | Pages 9-10, Lines 180-185 and 192-203, Section: Statistical analysis |
|  |  | (*b*) Describe any methods used to examine subgroups and interactions | Page 9, Lines 180-185, Section: Statistical analysis |
|  |  | (*c*) Explain how missing data were addressed | Page 10, Lines 208-210, Section: Basic sociodemographic characteristics of the participants |
|  |  | (*d*) *Cohort study*—If applicable, explain how loss to follow-up was addressed  *Case-control study*—If applicable, explain how matching of cases and controls was addressed  *Cross-sectional study*—If applicable, describe analytical methods taking account of sampling strategy | Page 7, Lines 130-140, Section: Sample size and sampling method |
|  |  | (*e*) Describe any sensitivity analyses | Pages 9-10, Lines 192-203, Section: Statistical analysis |

Continued on next page

| Results | | | Reported on Page No |
| --- | --- | --- | --- |
| Participants | 13* | (a) Report numbers of individuals at each stage of study—eg numbers potentially eligible, examined for eligibility, confirmed eligible, included in the study, completing follow-up, and analysed | Page 10, Line 208, Section: Basic sociodemographic characteristics of the participants |
|  |  | (b) Give reasons for non-participation at each stage | Page 10, Lines 208-210, Section: Basic sociodemographic characteristics of the participants |
|  |  | (c) Consider use of a flow diagram | The participant flow process is described on Page 7, Lines 130-140, in the section “Sample size and sampling method” and Page 10, Lines 208-210, in the section “Basic sociodemographic characteristics of the participants.” |
| Descriptive data | 14* | (a) Give characteristics of study participants (eg demographic, clinical, social) and information on exposures and potential confounders | Pages 10-11, Lines 208-219, Section: Basic sociodemographic characteristics of the participants  Page 9, Lines 180-185: Statistical analysis |
|  |  | (b) Indicate number of participants with missing data for each variable of interest | Page 10, Lines 208-210, Section: Basic sociodemographic characteristics of the participants |
|  |  | (c) *Cohort study*—Summarise follow-up time (eg, average and total amount) | *Not applicable - Cross-sectional study* |
| Outcome data | 15* | *Cohort study*—Report numbers of outcome events or summary measures over time | *Not applicable - Cross-sectional study* |
|  |  | *Case-control study—*Report numbers in each exposure category, or summary measures of exposure | *Not applicable - Cross-sectional study* |
|  |  | *Cross-sectional study—*Report numbers of outcome events or summary measures | Pages 11-12, Lines 235-242, Section: Unintentional home injury environmental risks for children aged 0–6 years  Table 1 and S1 Fig |
| Main results | 16 | (*a*) Give unadjusted estimates and, if applicable, confounder-adjusted estimates and their precision (eg, 95% confidence interval). Make clear which confounders were adjusted for and why they were included | Table 4 and Page 17, Lines 273-290, Section: Influencing factors of unintentional home injury environmental risk scores for children aged 0–6 years in the urban area |
|  |  | (*b*) Report category boundaries when continuous variables were categorized | Page 7, Lines 132-138, Section: Sample size and sampling method |
|  |  | (*c*) If relevant, consider translating estimates of relative risk into absolute risk for a meaningful time period | Pages 11-12, Lines 235-242, Section: Unintentional home injury environmental risks for children aged 0–6 years  Table 1 and S1 Fig |
| Other analyses | 17 | Report other analyses done—eg analyses of subgroups and interactions, and sensitivity analyses | Page 13-16, Lines 248-270, Section: Comparison of unintentional home injury environmental assessment scores among populations with different characteristics  Table 2 and 3 |
| Discussion | | |  |
| Key results | 18 | Summarise key results with reference to study objectives | Page 19, Lines 297-307, Section: Discussion |
| Limitations | 19 | Discuss limitations of the study, taking into account sources of potential bias or imprecision. Discuss both direction and magnitude of any potential bias | Page 25, Lines 420-429, Section: Limitations |
| Interpretation | 20 | Give a cautious overall interpretation of results considering objectives, limitations, multiplicity of analyses, results from similar studies, and other relevant evidence | Page 19-23, Lines 309-389, Sections:   - Types of main caregivers and incidence rates of unintentional injuries among children aged 0–6 years in urban areas - Home environments of children aged 0–6 years in urban areas require improvement - Influencing factors of unintentional home injury environmental risks for children aged 0–6 years in the urban area |
| Generalisability | 21 | Discuss the generalisability (external validity) of the study results | Page 23, Lines 392-417, Section: Recommendations for enhancing home environment safety  Page 25, Lines 432-437, Section: Significance |
| Other information | | |  |
| Funding | 22 | Give the source of funding and the role of the funders for the present study and, if applicable, for the original study on which the present article is based | Page 27, Lines 460-466, Section: Financial disclosure statement |

*Give information separately for cases and controls in case-control studies and, if applicable, for exposed and unexposed groups in cohort and cross-sectional studies.

**Note:** An Explanation and Elaboration article discusses each checklist item and gives methodological background and published examples of transparent reporting. The STROBE checklist is best used in conjunction with this article (freely available on the Web sites of PLoS Medicine at http://www.plosmedicine.org/, Annals of Internal Medicine at http://www.annals.org/, and Epidemiology at http://www.epidem.com/). Information on the STROBE Initiative is available at www.strobe-statement.org.
